# Stress Granules and Acute Ischemic Stroke: Beyond mRNA Translation

**DOI:** 10.3390/ijms23073747

**Published:** 2022-03-29

**Authors:** Marta Aramburu-Núñez, Antía Custodia, María Pérez-Mato, Ramón Iglesias-Rey, Francisco Campos, José Castillo, Alberto Ouro, Daniel Romaus-Sanjurjo, Tomás Sobrino

**Affiliations:** 1 NeuroAging Group (NEURAL), Clinical Neurosciences Research Laboratory (LINC), Health Research Institute of Santiago de Compostela (IDIS), 15706 Santiago de Compostela, Spain; marta.aramburu.nunez@sergas.es (M.A.-N.); antia.custodia.malvido@sergas.es (A.C.); tomas.sobrino.moreiras@sergas.es (T.S.); 2 Neurological Sciences and Cerebrovascular Research Laboratory, Department of Neurology and Stroke Center, La Paz University Hospital, Neuroscience Area of IdiPAZ Health Research Institute, Universidad Autónoma de Madrid, 28046 Madrid, Spain; maria.perez.mato@sergas.es; 3 Neuroimaging and Biotechnology Laboratory (NOBEL), Clinical Neurosciences Research Laboratory (LINC), Health Research Institute of Santiago de Compostela (IDIS), 15706 Santiago de Compostela, Spain; ramon.iglesias.rey@sergas.es (R.I.-R.); jose.castillo.sanchez@sergas.es (J.C.); 4 Translational Stroke Laboratory Group (TREAT), Clinical Neurosciences Research Laboratory (LINC), Health Research Institute of Santiago de Compostela (IDIS), 15706 Santiago de Compostela, Spain; francisco.campos.perez@sergas.es

**Keywords:** endothelial cell, eukaryotic initiation factor 2, eukaryotic initiation factor 4F, hippocampus, ischemia, neuron, stress granules, stroke

## Abstract

Ischemic stroke is a leading cause of death and disability worldwide. Following an ischemic insult, cells undergo endoplasmic reticulum (ER) stress, which increases the ER’s protein-folding and degradative capacities and blocks the global synthesis of proteins by phosphorylating the eukaryotic translation initiation factor 2-alpha (eIF2α). Phosphorylation of eIF2α is directly related to the dynamics of stress granules (SGs), which are membraneless organelles composed of RNA-binding proteins and mRNA. SGs play a critical role in mRNA metabolism and translational control. Other translation factors are also linked to cellular pathways, including SG dynamics following a stroke. Because the formation of SGs is closely connected to mRNA translation, it is interesting to study the relationship between SG dynamics and cellular outcome in cases of ischemic damage. Therefore, in this review, we focus on the role of SG dynamics during cerebral ischemia.

## 1. Introduction

Ischemic stroke remains one of the leading causes of mortality and disability in Europe [1]. Following an ischemic insult, two large areas can be distinguished: the ischemic core, infarcted tissue with irreparable damage; and the penumbra area, surrounding the ischemic core, which contains hypoperfused tissue that is still viable [2]. Therefore, avoiding the transformation of penumbral into infarcted tissue is a key step in overcoming neuronal damage, as well as improving outcomes in patients. Following the occlusion of a blood vessel, the pathological paths of the ischemic cascade are activated, which eventually leads to neuronal death by necrosis or apoptosis [3]. A hypoxic environment triggers glutamate excitotoxicity through a massive release of glutamate after injury, which causes neurotoxicity by binding to N-methyl-D-aspartate receptors (NMDAR), thereby promoting the entry of large amounts of Ca^2+^ into neurons [3]. High concentrations of intracellular Ca^2+^ produce neuronal damage by increasing endoplasmic reticulum (ER) stress and the number of reactive oxygen species (ROS), and by causing a depletion of ATP-dependent processes [3].

**Figure 1:**
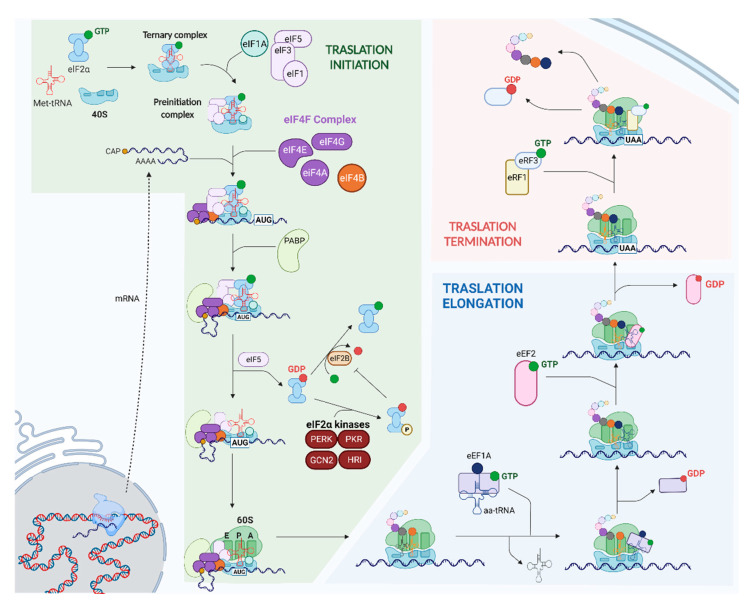
Assembly of the preinitiation complex (PIC), eIF4F complex (eIF4E, eIF4G, and eIF4A), and eIF4B in the 5′ cap of the mRNA triggers initiation (dashed arrow). Once a start codon (AUG) is found, eIF2-GDP is released, the 60S ribosomal subunit joins the PIC, and an elongation-competent 80S ribosome is formed. eIF2-GDP can undergo incapacitating phosphorylation that blocks protein synthesis under stress conditions. During elongation, the ternary complexes of eEF1A-GTP-aminoacyl-tRNA bind to the ribosome at the A site, increasing the length of the aminoacidic chain. Several rounds of elongation occur until a stop codon is reached, at which point the complex eRF1-eRF3-GTP recognizes it and translation is terminated.

The translation of protein-coding nuclear genes is a vital cytoplasmic process necessary for development and cellular homeostasis [4,5] (Figure 1). The initiation of translation is a highly regulated process that begins with the recruitment of the 40S small ribosomal subunit and the ternary complex (TC), composed of the initiator methionyl-tRNA and the GTP-linked eukaryotic initiation factor 2 (eIF2α) [5,6]. Then, the 40S ribosome and the TC bind to several eukaryotic initiation factors, such as eIF1, eIF1A, eIF5, and eIF3, to form the preinitiation complex (PIC), which is recruited to the 5′ cap of the mRNA by the heterotrimeric eIF4F complex (consisting of the cap-binding protein eIF4E, RNA helicase eIF4A, and scaffold protein eIF4G) and eIF4B [5,6]. Thereafter, the poly(A)-binding protein (PABP) promotes the circularization of the mRNA and allows for the scanning of the PIC in a 5′ to 3′ direction, which eventually pauses at a start codon (AUG) [5,7]. This recognition triggers the hydrolysis of eIF2-GTP to eIF2-GDP (catalyzed by the GTPase activating protein eIF5), as well as other series of events that culminate in the recruitment of the large 60S ribosomal subunit and the formation of an elongation-competent ribosome [5,6] (Figure 1). Next, aminoacyl-tRNAs are carried to the A site of the ribosome in a GTP-dependent manner by eukaryotic elongation factors (eEF1As) during the elongation phase of translation [4,5,8]. Following codon recognition by the tRNA, eEF1A-GDP is released and the polypeptide is transferred from the previous tRNA in the P site to the aminoacyl tRNA in the A site, extending the nascent chain [4,5]. The binding of the GTP-bound eEF2 onto the A site is necessary for a correct translocation of the ribosome along the mRNA [4,5]. After that, the deacylated tRNA is released from the E site, with the peptidyl-tRNA placed in the P site, and the free A site awaits the next aminoacyl tRNA [4,5] (Figure 1). Finally, the protein translation process finishes when the elongating ribosome reaches a stop codon in the A site (UAA, UAG, or UGA), triggering the termination of translation. This stop codon is recognized by the eukaryotic release factor 1 (eRF1)-eRF3-GTP complex, which allows for the release of the polypeptide [4,5] (Figure 1).

**Figure 2:**
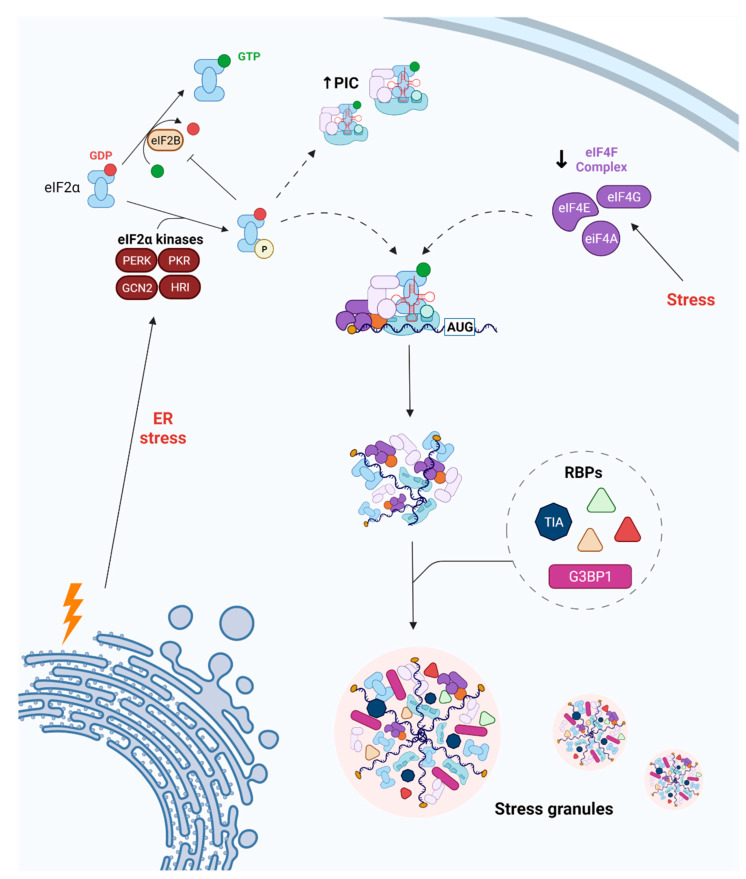
The phosphorylation of eIF2α and disassembly of the eIF4F complex are the main pathways blocking protein translation in cases of endoplasmic reticulum (ER) stress. The kinases PERK, PKR, GCN2, and HRI can phosphorylate and, therefore, inhibit eIF2α. In both cases, RNA-binding proteins (RBPs) such as TIA1 or G3BP1, among others, interact with the incomplete PIC and promote SG formation (dashed arrows). Importantly, the nucleated SGs mature by adding other RBPs that attach to existing mRNAs and/or bring new mRNAs.

It is well-established that ER stress plays an important role in the pathophysiology and outcome of ischemic stroke [9]. ER stress induced by ischemic injury triggers the phosphorylation of eIF2α to block the global synthesis of proteins and, subsequently, to increase the protein-folding and degradative capacities of the ER [10,11]. So far, four kinases have been described as able to phosphorylate eIF2α, with a major role played by EIF2AK2 (PKR) and EIF2AK3 (PERK) in the ischemic event [11,12] (Figure 2). Although the phosphorylation of eIF2α decreases the global rate of protein synthesis, some mRNAs are translated even during ischemia in order to cope with ER stress, including the activating transcription factor ATF4 and the nuclear factor erythroid 2-related factor 1 (NFE2L1) [13,14,15].

ER-stress-mediated eIF2α phosphorylation may induce the temporary formation of stress granules (SGs), which are cytoplasmic membraneless organelles composed of translationally arrested PICs, mRNAs, and associated translation initiation factors [16,17,18] (Figure 2). In addition to eIF2α phosphorylation, SG formation can be also triggered by inactivating the eIF4F complex [16] or silencing eRF1 [19] (Figure 2); thus, the formation of SGs is closely connected to the translational status in the cell [18]. Moreover, RNA-binding proteins (RBPs), such as the T-cell intracellular antigen 1 (TIA1) and the RAS GTP-activating protein-binding protein 1 (G3BP1), interact with SGs and mediate their quick assembly and disassembly by binding directly to mRNAs and translational machinery, and/or by interacting with PICs [18]. These RBPs are linked to untranslated transcripts that can oligomerize (SG core) and ‘seed’ to other SG cores composed of different RBPs, finally forming ‘shell’-like structures around them [18]. This dynamic nature is the most remarkable feature of SGs, allowing for a fast response (assembly) when a stress is presented, as well as a fast disassembly when the stress is gone [18]. An imbalance with regard to SG assembly underlies many cellular mechanisms involved in neurodegeneration [16,18].

Healthy neurons express many components of SGs, which can be visibly detectable as granules or present in subdetectable forms. Emerging findings show that SGs can assemble in both the cell bodies and axonal compartments and exhibit dynamic changes in response to damage [16]. SGs may have evolved in order for cells to respond adaptively to transient challenges, but under chronic conditions, persistent SGs are considered a potential trigger for cell death. Recently, SG formation has been hypothesized to be a central downstream event in the development of several neurodegenerative disorders, including amyotrophic lateral sclerosis, frontotemporal dementia, and Alzheimer’s disease [16]. Below, we review the available literature assessing the role of SG dynamics in both in vitro and in vivo models of cerebral ischemia.

## 2. SG Dynamics following Cerebral Ischemia

### 2.1. Changes in RBP Expression

Recently, proteomics and immunohistochemical studies have shed light on the dynamics of several actors that mediate SG assembly after cerebral ischemia [20,21]. Cortical protein aggregations of RBPs, such as heterogeneous nuclear ribonucleoproteins A0 and A1 (hnRNPA0 and hnRNPA1, respectively), heterogeneous nuclear ribonucleoprotein P2 (FUS), and TAR DNA-binding protein 43 (TDP-43), were found in a mouse model of cerebral ischemia induced by transient middle cerebral artery occlusion (tMCAO) [20]. Importantly, these aggregations of hnRNPA0, hnRNPA1, FUS, and TDP-43 were present at 1 h after reperfusion, but dissolved after 24 h [20]. All RBP aggregations were irreversible when a permanent MCAO model was used [20]. This seems to indicate that reperfusion is needed to dissolve RBP aggregations [20]. Increased levels of nuclear and cytoplasmic TDP-43 were found, with a peak at 48 h post-injury, in the neurons and glia of rats with subarachnoid hemorrhages [22]. Similarly, another study using the tMCAO mouse model observed that the immunoreactivity of TDP-43, FUS, and hnRNPA1 is subject to similar dynamics [21]. Specifically, there was an increase in all RBPs at 1, 6, and 24 h of reperfusion compared with sham animals; the expression of all RBPs started to decrease to sham levels at 24 h of reperfusion [21]. The subcellular expression of all RBPs in sham animals was predominantly localized in the nuclei of both neurons and astrocytes. However, this pattern changed only in neurons from ischemic animals, where TDP43, FUS, and hnRNPA1 mainly displayed cytoplasmic expression [21]. In summary, although neither study directly assessed the relationship between these RBPs and SG formation, a link between them is plausible [23,24,25,26]. Moreover, hnRNPA0, hnRNPA1, TDP-43, and FUS are translocated from the nucleus to the cytoplasm, where it is hypothesized that they form SGs to protect mRNAs during ischemia. Once oxygen flux is restored, these SGs are disassembled and RBPs return to the nucleus. However, if this stress stimulus is maintained over time (as is the case with a permanent MCAO or hemorrhagic stroke), there is no insolubility of RBPs and, thus, no SG disassembly allowing translation to restart; eventually, this causes neuronal death by apoptosis.

An abnormal cytoplasmic accumulation of RBPs may underlie cellular pathways related to neurodegeneration. Thammisetty et al. demonstrated that the aging-mediated accumulation of cytoplasmic TDP-43 leads to modifications that make it a dysfunctional protein [27]. The higher cytoplasmic immunoreactivity of truncated TDP-43 was correlated with a larger ischemic lesion, increased neuronal death, and enhanced inflammation at 72 h post-reperfusion in older mice compared with younger ones, both groups having tMCAOs [27]. In addition, another study using a rat model of transient focal cerebral ischemia found that damaged neurons displayed cytoplasmic translocation of TDP-43 [28]. TDP-43 was mainly colocalized with ubiquitin granules, showing higher levels of apoptosis after 24 h of reperfusion [28]. Overall, the inability of TDP-43 to return to the nucleus triggers the creation of pathogenic forms of TDP-43 that partially underlie a worse outcome following ischemia, even when blood supply is restored. As for other chronic neurodegenerative diseases [29], aging appears to have a key role in the outcome following ischemia by further increasing SG-mediated neuronal death.

Different RBPs neither have to respond in the same way nor have the same role in neuronal outcome following an ischemic insult. For example, hnRNPA2-B1 is another RBP whose immunohistochemical signal globally decreased after 3, 6, 12, and 24 h of reperfusion in a rat tMCAO model [30]. Similar to other RBPs, this reduction was also accompanied by a cortical, but not hippocampal, translocation of hnRNPA2-B1 from the nucleus to the cytoplasm during reperfusion [30]. A higher level of such translocation was seen in the more severely damaged cortical areas compared with the other cortical areas observed [30]. Therefore, each RBP confers different characteristics to SGs, and this probably affects their assembly and disassembly, eventually impacting neuronal death.

To summarize, RBPs are moved from the nucleus to the cytoplasm right after the onset of cerebral ischemia; there, they trigger the formation of SGs and protect mRNAs from ischemic events. Reperfusion seems to be a key step for RBPs to return to the nucleus, which allows for the restoration of protein synthesis once the ischemia is resolved. A lack of restoration of blood flux causes severe neuronal damage by promoting prolonged translation arrest. Considering their implications in the relevant pathophysiologies, both processes are good candidates with which to find either biomarkers or therapeutic targets following ischemic stroke.

### 2.2. Modulation of RBP Function and Expression

The RNA-binding protein motif 3 (RBM3) is a well-known cold shock protein with beneficial roles following a stroke in both animal models and humans [31,32]. Recently, the interaction between RBM3 and G3BP1 was suggested to mediate the survival of both the PC12 cell line and rat primary cortical neuronal cultures in an in vitro model of ischemia [33]. In particular, RBM3–G3BP1 union might increase the ratio of SGs by facilitating their nucleation and formation, leading to a reduction in the rate of cell death [33]. Another RBP related to SGs, called argonaute RISC catalytic component 2 (AGO2), was found to colocalize with G3BP1 [34]. AGO2, a protein mainly involved in microRNA (miRNA) maturation, was described as interacting with G3BP1 in the cytoplasm of microglial cells undergoing in vitro ischemia [34]. Therefore, we can assume that these RBPs trigger conformational changes of G3BP1 in a way that facilitates the formation of the SG core and/or promotes the synthesis of miRNAs mediating RBP dynamics. In any case, such interactions seem to provide cell protection during times of ischemic stress.

Several studies have focused on the role of miRNAs (short noncoding RNAs that suppress the expression of mRNAs) regarding SG dynamics [19,35,36]. Si et al. observed a reduced rate of SG formation while at the highest level of apoptosis and infarct volume, 24 h after reperfusion in tMCAO rats [35]. Following tMCAO, miRNA-335 was the most downregulated miRNA in the damaged areas; its forced overexpression led to a rise in the rate of SG formation, a reduced infarct volume, and decreased neuronal apoptosis [35]. miRNA-335 blocked the Rho-associated protein kinase 2 (ROCK2) and thus decreased the phosphorylation of TIA1 [35]. Notably, miRNA-335 was suggested as an early diagnostic biomarker for stroke patients [35]. Furthermore, it was also discovered that miRNA-335 can target eRF1 and promote SG formation in vitro [19]. Specifically, methyltransferase 3 (METTL3) adds a m6A modification to the primary miRNA-335, thereby increasing its production, which eventually reduces the protein expression of eRF1 [19]. Thus, miRNA-335 acts on both ROCK2 and eRF1 to promote SG assembly, leading to neuronal survival under ischemic conditions. In addition, miRNAs can directly target RBPs and block them in order to enhance cellular survival. For instance, miRNA-451 promoted cell survival and reduced oxidative stress in PC12 cells in an in vitro ischemia model by blocking the expression of the RBP known as CUGBP Elav-like family member 2 (CELF2) [36]. 

In summary, these findings suggest that different actors can interact and modify RBPs so that their features related to SG dynamics can be altered, which eventually leads to different outcomes following ischemic injury. Therefore, the complexity of SG dynamics is not only based on what triggers SG assembly (eIF2α phosphorylation, dysfunctional eIF4F, or eRF1 silencing), but also on the dynamics and characteristics of each RBP.

### 2.3. Endothelial Cells and SG Dynamics

The blood–brain barrier (BBB) is a cellular structure that acts as a barrier between the blood and the central nervous system, participating in its protection and homeostasis. The BBB is composed of endothelial cells, pericytes, an astrocyte end-foot, and a basement membrane [37,38]. Following ischemic stroke, a short disruption of the BBB occurs, which eventually generates brain damage [38]. Furthermore, similar to neurons and glia, the ischemic insult induces ER dysfunction in endothelial cells and pericytes, with a subsequent reduction in translation initiation and induction of SG formation [16,39].

Currently, the data regarding SG dynamics in endothelial brain cells after stroke are limited. Only the relationship between TDP-43 and stroke was studied [39]. Endothelial cells from mice with tMCAOs displayed increased levels of TDP-43, mostly located in the cytoplasm at 24 h following the onset of ischemia and returning to the nucleus at 48 h [39]. In addition, in vitro experiments revealed that the accumulation of TDP-43 in the cytoplasm of endothelial cells led to a reduction in the number of tight junctions and the inhibition of their migration capacity (negatively affecting BBB integrity and permeability); although it also increased their proliferation (promoting BBB repair) [39]. Given that TDP-43 is translocated earlier to the cytoplasm and is involved in SG formation [23], it can be hypothesized that TDP-43 acts in a similar way in endothelial cells following ischemia. Although this first study presented both harmful and beneficial outcomes, more studies are needed in order to better elucidate the mechanism/s underlying SG formation and vascular outcome after stroke. No publications were found regarding the presence of SGs in pericytes after stroke. As previously mentioned, this is a new field to investigate, as information on SGs, strokes, and the BBB is extremely scarce or non-existent.

## 3. Two Different Ways SG Dynamics Act in Triggering Neuroprotection after Ischemia 

In the 1980s, it was observed that, under ischemic conditions, neurons from the hippocampal CA1 region undergo an irreversible inhibition of translation, which results in neuronal death; this is in contrast to resilient CA3 neurons [40,41,42]. Several studies have highlighted the relationship between SGs and the different survival outcomes seen in CA1 and CA3 neurons [24,25,26]. Following 10 min of cardiac arrest and a subsequent 10 min of reperfusion, there was an increase in SG assembly (assessed by cytoplasmic colabeling of TIA1 and S6, a marker of the small ribosomal subunit 40S) in rat CA3 neurons, but not in the CA1 region [26]. This value for CA3 neurons returned to normal conditions after 90 min of reperfusion, whereas CA1 neurons showed a later colabeling of TIA1 and S6 at 4 h post-reperfusion [26]. This SG assembly was not triggered by eIF2α phosphorylation, but by a negative altering of the eIF4F complex [24]. Given that SGs are sites of inhibited translation, it is feasible that the later SG assembly seen in CA1 neurons underlies their irreversible inhibition of translation [26]. An in vitro study, using the hippocampal neuronal cell line HT22, reported that the inability of TIA1 to interact with its mRNA targets provokes apoptosis under oxidative stress [43]. Accordingly, the oxidation of TIA1 may interfere with its protective role of binding mRNAs and promoting temporary SG assembly through a reduction in housekeeping protein synthesis under stress conditions [43]. Another in vivo study showed a hypothetical mechanism connecting SGs and the delayed death of CA1 neurons [25]. The immunohistochemical coexpression of SGs (TIA1 + S6 co-labeling) and ubiquitin-labeled protein aggregates was found in the CA1, but not the CA3, neurons of rats undergoing bilateral carotid artery occlusion after 2 days of reperfusion [25]. Therefore, it was suggested that these protein aggregates impair SG dynamics by impeding their disassembly [25]. This event could be related to the delayed translation of the heat shock chaperone 70 (Hsp70), detected in CA1 neurons but not CA3 neurons [25,44]. Notably, the preischemia application of cycloheximide (a drug that prevents SG assembly) was able to increase the survival of CA1 neurons after 3 days of reperfusion [24].

The TIA-1-related protein (TIAR) is another RBP involved in the formation of SGs, which was suggested to share redundant functions with TIA1 to some extent [45]. Accordingly, a significant increase in the expression of hippocampal and cortical TIAR was observed following transient global ischemia, by ligation of both carotid arteries and tMCAO in rats, respectively [46]. SGs containing TIAR and TIA1 negatively target the mRNA of the hypoxia-inducible factor 1α (HIF-1α), one of the main transcription factors involved in the response to hypoxia stress [45,47,48]. Mice with a targeted knock-out mutation for HIF-1α in hippocampal and cortical neurons showed a reduction in neuronal death at 72 h post-reperfusion compared with control mice following two different ischemic conditions: ambient hypoxia and bilateral common carotid artery occlusion [48]. 

Overall, it seems that the dysregulation of the equilibrium between SGs and translating polysomes partially underlies the delayed death of CA1 neurons. We hypothesize that the ischemia-mediated inability of TIA1 and/or delay in the formation of TIA1-assembled SGs may impede the protection of translating mRNAs under stress conditions in CA1 neurons. These events, along with the interaction of SGs with ubiquitinated protein aggregates, lead to irreversible arrested translation and, eventually, neuronal death. In contrast, the cycloheximide-induced trapping of mRNAs onto polysomes during ischemia inhibits SG assembly and their subsequent binding to protein aggregates, which results in higher CA1 neuron survival. However, the ‘kidnapping’ of some mRNAs such as HIF-1α is also necessary to promote neuroprotection under ischemic conditions. Therefore, these studies highlight the necessity of assessing the different roles of other RBPs in the formation of SGs, as well as their relationship to ischemia-mediated cellular pathways, as they are clearly involved in neuronal fate following damage.

## 4. Concluding Remarks

The study of SG dynamics during ischemia has the potential to produce some of the most promising results with regard to stroke conditions. However, it has not been fully explored yet and there are many gaps that remain to be tackled. The scarcely available literature assessing SG dynamics under several ischemic conditions (in vitro and different in vivo approaches) aims to provide a general idea of the neuroprotective role of SGs during the acute phase of ischemia. Once the ischemic insult begins, the faster assembly of SGs may underlie the higher rates of neuronal survival by protecting housekeeping mRNAs and/or blocking harmful mRNAs. The translocation of RBP-forming SGs from the nucleus to the cytoplasm is reduced and RBPs then return to the nucleus when blood supply is restored. However, prolonged ischemia maintains RBPs in the cytoplasm, which may cause severe neuronal damage by either keeping protein translation blocked or allowing for the union of protein aggregates or ubiquitinating RBPs. These conditions appear to be affected by aging, which further increases the interest in studying SG dynamics during stroke, as it is a usual condition among the elderly. Furthermore, the complexity of SG dynamics increases when other RBPs and miRNAs, either positively or negatively, interact with RBPs that assemble SGs. Regarding future clinical applications, some key factors in SG dynamics could be used as early biomarkers or even as therapeutic targets. Therefore, the study of the different molecules implicated in SG dynamics may have implications on a basic molecular level but also in the clinical application of such studies in stroke patients. 

In summary, SG dynamics in cases of stroke is an open field that demands more research in order for us to fully understand and decipher the mechanisms underlying the different connections between RBPs, mRNAs, and cellular translation states. In the future, SG dynamics may offer therapeutic targets as disease-evolution biomarkers for patients suffering from a stroke.
